# KSP inhibitor ARRY-520 as a substitute for Paclitaxel in Type I ovarian cancer cells

**DOI:** 10.1186/1479-5876-7-63

**Published:** 2009-07-20

**Authors:** Ki Hyung Kim, Yanhua Xie, Ewan M Tytler, Richard Woessner, Gil Mor, Ayesha B Alvero

**Affiliations:** 1 Department of Obstetrics, Gynecology and Reproductive Sciences, Yale University School of Medicine, New Haven, CT, USA; 2 Department of Obstetrics and Gynecology, Pusan National University, Busan, Korea; 3 Department of Surgery, University of Alabama, Birmingham, AL, USA; 4 Department of Pharmacology, Array BioPharma, Boulder, CO, USA

## Abstract

**Background:**

We previously described a sub-population of epithelial ovarian cancer (EOC) cells with a functional TLR-4/MyD88/NF-κB pathway (Type I EOC cells), which confers the capacity to respond to Paclitaxel, a known TLR-4 ligand, by enhancing NF-κB activity and upregulating cytokine secretion – events that are known to promote tumor progression. It is therefore important to distinguish those patients that should not receive Paclitaxel; it is also important to identify alternative chemotherapy options that would benefit this sub-group of patients. The objective of this study is to determine if the KSP inhibitor, ARRY-520, can be a substitute for Paclitaxel in patients with Type I EOC.

**Methods:**

EOC cells isolated from either ascites or tumor tissue were treated with increasing concentrations of ARRY-520 or Paclitaxel and cell viability determined. Activation of the apoptotic pathway was determined using Western blot analysis. Mitochondrial integrity was quantified using JC1 dye. Cytokine profiling was performed from supernatants using xMAP technology. NF-κB activity was measured using a Luciferase reporter system. *In vivo *activity was determined using a subcutaneous xenograft mouse model.

**Results:**

ARRY-520 and Paclitaxel exhibited the same cytotoxic effect on Type I and II cells. The GI_50 _at 48 h for Type II EOC cells was 0.0015 μM and 0.2 μM for ARRY-520 and Paclitaxel, respectively. For Type I EOC cells, the GI_50 _at 48 h was > 3 μM and >20 μM for ARRY-520 and Paclitaxel, respectively. Decrease in the number of viable cells was accompanied by mitochondrial depolarization and caspase activation. Unlike Paclitaxel, ARRY-520 did not induce NF-κB activation, did not enhance cytokine secretion, nor induce ERK phosphorylation in Type I EOC cells.

**Conclusion:**

Administration of Paclitaxel to patients with high percentage Type I cancer cells could have detrimental effects due to Paclitaxel-induced enhancement of NF-κB and ERK activities, and cytokine production (e.g. IL-6), which promote chemoresistance and tumor progression. ARRY-520 has similar anti-tumor activity in EOC cells as that of Paclitaxel. However, unlike Paclitaxel, it does not induce these pro-tumor effects in Type I cells. Therefore, the KSP inhibitor ARRY-520 may represent an alternative to Paclitaxel in this subgroup of EOC patients.

## Background

Epithelial ovarian cancer (EOC) is the fifth leading cause of cancer-related deaths in women and is the most lethal of the gynecologic malignancies [1]. The standard of care for newly diagnosed EOC patients is surgical debulking and administration of a platinum and taxane -based chemotherapy regimen, usually carboplatin and paclitaxel, given either as neo-adjuvant or adjuvant therapy. With this regimen, 80–90% will initially respond but less than 10–15% will remain in complete remission [2,3]. The percentage of non-responders increases significantly to 65–75% for recurrent cancers[3]. Additionally, some patients progress during or shortly after completion of chemotherapy.

Recurrent ovarian cancer is characterized by chemoresistance to prior treatments, most commonly to Paclitaxel. Previously, we described the identification of a sub-population of EOC cells that are resistant to this agent. This sub-group of cells (Type I EOC cells) has a functional Toll Like Receptor-4-Myeloid Differentiation Protein 88- Nuclear factor κB (TLR-4/MyD88/NF-κB) pathway, and the ligation of TLR-4 by Paclitaxel (a known TLR-4 ligand) is able to induce NF-κB activation and secretion of pro-inflammatory and pro-tumor cytokines IL-6, IL-8, MCP-1, and GRO-α [4,5]. This response confers resistance to apoptosis, and more importantly, enhances tumor growth [4]. In contrast, these events were not observed in the group of EOC cells that did not have a functional TLR4-MyD88 pathway (Type II EOC cells) and are sensitive to Paclitaxel.

The treatment of Type I EOC cells with Paclitaxel is not only ineffective in killing these cells, but more importantly, can be detrimental since it may enhance tumor growth. Therefore, the identification of potential new therapies for this specific cell population would be beneficial for the treatment of ovarian cancer patients.

ARRY-520 is an inhibitor of the mitotic kinesin, KSP. KSP inhibition prevents bipolar spindle formation leading to mitotic arrest and cell death [6]. In studies comparing ARRY-520 with some of the more clinically advanced compounds and standard of care agents, ARRY-520 was shown to have superior efficacy in multiple xenograft models [7] and is currently in a Phase I trial [8]. More importantly, since KSP is expressed predominantly in proliferating cells and is absent from post-mitotic neurons, KSP inhibitors do not induce peripheral neuropathy usually observed with traditional microtubule disrupting agents such as Paclitaxel [9]. The objective of this study is two-fold. First, to determine and characterize the anti-tumor activity of the KSP-inhibitor, ARRY-520, in EOC cells; and second, to determine whether it is effective against Type I EOC cells and therefore could be used as a substitute for Paclitaxel.

We demonstrate that ARRY-520 is able to promote cell death in EOC cells through an apoptosis mediated mechanism, involving caspase-2 activation. More importantly, we showed that contrary to Paclitaxel, ARRY-520 has no effect on the TLR4 pathway and does not induce the secretion of pro-inflammatory and pro-tumor cytokines in Type I EOC cells.

## Methods

### Cell lines and culture conditions

Established human EOC cell lines, A2780 and A2780/CP70 (gifts from Dr. TC Hamilton) [10] were propagated in RPMI plus 10% fetal bovine serum (Gemini Bio-Products, Woodland, CA). Primary EOC cell lines were isolated from malignant ovarian ascites or explanted from ovarian tumors and cultured as previously described [11-13]. Use of patient material was approved by Yale University's Human Investigations Committee (HIC # 10425).

### Cell viability assay

Cell viability was determined as previously reported [12] using CellTiter 96^® ^AQueous One Solution Cell Proliferation Assay (Promega Corporation, Madison, WI). ARRY-520 (Array Biopharma, Boulder, CO) and Paclitaxel (Sigma Alrich) were added to the medium from a 10 μM and 3.8 mM stock, respectively to give various final concentrations as described in the results section. Each experiment was done in triplicate.

### Caspase-3/7, -8, and -9 activity assay

Caspase activity was measured using Caspase-Glo™ 3/7, 8, or 9 reagents (Promega) as previously described [12].

### SDS-PAGE and Western blots

SDS-PAGE and western blots were performed as previously described [12]. The following antibodies were used: mouse anti-caspase-2 (BD, 1:1,000), rabbit anti-Bid (Cell Signaling, Beverly, MA, 1:5,000), mouse anti-XIAP (BD, 1:1,000), mouse anti-phosphorylated ERK (Santa Cruz Biotechnology, 1:200), and rabbit anti-actin (Sigma, 1:10,000).

### Assay of mitochondrial depolarization using JC-1

Cells were trypsinized and stained with JC-1 dye using the Mitocapture™ mitochondrial apoptosis detection kit (BioVision Research Products, Mountain View, CA) according to manufacturer's instructions. Data was acquired using FACS Calibur System and analyzed using CellQuest software (BD Biosciences, San Jose, CA).

### Assay for NF-κB activity

NF-κB activity was measured using a luciferase reporter construct, pBII-LUC, containing two κB sites before a Fos essential promoter (a gift from Dr. S. Ghosh, Yale University) [5]. Cells were transiently transfected using the FuGENE 6 Transfection Reagent (Roche Applied Science, Indianapolis, IN) following the manufacturer's instructions. Luciferase activity was measured using the Luciferase Assay System (Promega, Madison, WI) according to the manufacturer's protocol. Briefly, 10 μg of each protein sample in a total volume of 100 μl was mixed with 20 μl of the Luciferase Assay Reagent, and luminescence measured using a TD 20/20 Luminometer (Turner Designs, Sunnyvale, CA). Relative activity was calculated based on readings measured from untreated cells after subtracting blank values. Baseline was set to 100 units. Each sample was measured in triplicate.

### Cytokine profiling

Cytokines were measured from culture supernatants using the Bio-Plex system (Bio-RAD, Hercules, CA) as previously described [5,11,14,15].

### Mouse xenograft model

The Institutional Animal Care and Use Committee in Array Biopharma approved all *in vivo *work. Subcutaneous tumors were established in female nude mice using A2780 and a primary culture of EOC cells isolated from ascites. For each model, mice were randomized into six groups (n = 8). Group 1: saline (vehicle for ARRY-520); Group 2: 10% cremophor, 10% ethanol (vehicle for Paclitaxel); Group 3: 20 mg/kg ARRY-520; Group 4: 30 mg/kg ARRY-520; Group 5: 20 mg/kg Paclitaxel; and Group 6: 30 mg/kg Paclitaxel. Vehicle and compounds were administered IP, q4dx3. This treatment schedule was chosen based on previous anti-tumor and toxicology studies [15-17]. Tumor size was measured twice a week.

## Results

### ARRY-520 is cytotoxic in Type II EOC cells

**Figure 1:**
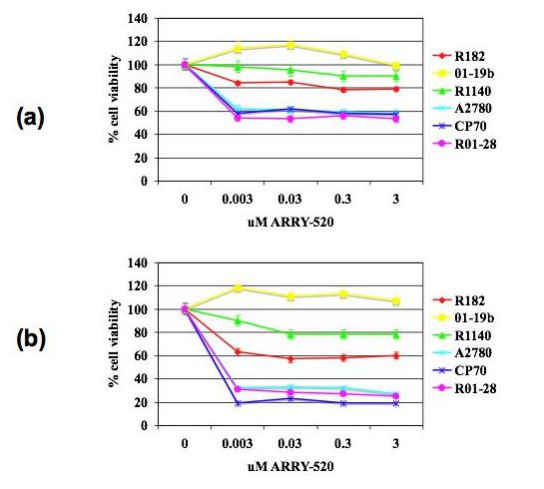
**ARRY-520 significantly decreases the number of viable Type II EOC cells**. The viability (in percentage, normalized to untreated cells) of EOC cells after treatment with increasing concentrations of ARRY-520 for (a) 24 and (b) 48 hours. Data were compiled from at least three independent experiments, each done in triplicate. Type I cells – R182, 01–19b, R1140; Type II cells – A2780, CP70, 01–28; dotted line corresponds to 50% viability.

Our first objective was to determine the effect of ARRY-520 on EOC cells. Thus, two established EOC cell lines (A2780, CP70) and four EOC cell cultures isolated from malignant ovarian ascites (R182, 01–28, 01–19b, R1140) were treated with increasing concentrations of ARRY-520 (up to 3 μM) or Paclitaxel (up to 20 μM) for 24 and 48 hours and cell viability was determined using the CellTiter 96 AQueous One Solution Cell Proliferation Assay. ARRY-520 effectively decreased cell viability in a time-dependent manner in the Type II EOC cell lines A2780, CP70, and 01–28 but had minimal effect on Paclitaxel-resistant Type I EOC cell lines R182, 01–19b, and R1140 (Fig. 1A–B). In Type II cell lines, the most prominent effect on cell viability was observed following 48 hours of treatment, with 50% growth inhibition (GI_50_) observed at 1.5 nM. At the same time-point, the GI_50 _for Type I cells was > 3,000 nM. Interestingly, we saw a similar pattern of response with equivalent pharmacologic doses of Paclitaxel. As shown in Table 1, GI_50 _was not reached in either compound in Type I EOC cells.

**Table 1:** *In Vitro *Response of EOC Cells

**Cell line**	**GI_**50 **_for** **ARRY-520, μM**	**GI_**50 **_for** **Paclitaxel, μM**
A2780	0.0015	0.2
CP70	0.0015	0.2
01–28	0.0015	0.2
R182	>3	>20
01–19b	>3	>20
R1140	>3	>20

### ARRY-520 induces apoptosis in Type II EOC cells

**Figure 2:**
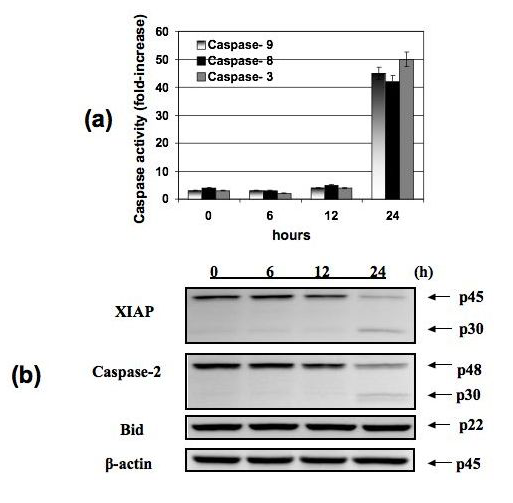
**ARRY-520 induces apoptosis in Type II EOC cells**. Type II EOC cells were treated with 3 μM ARRY-520 for 6, 12, and 24 hours. "0" designation represents non-treated controls. (a) Activity of capase-3, -8, and -9 was measured using Caspase-Glo assay, and (b) effect on XIAP, Caspase-2, and Bid was determined using Western blot analysis. Results shown are for CP70. Similar results were observed with other cells tested.

To determine whether the decrease in cell viability is due to the induction of apoptosis, we measured caspase activity in ARRY-520-treated Type II EOC cells. Following ARRY-520 treatment, a significant increase in the activity of caspases- 8, 9, and 3 was observed in a time-dependent manner (Fig. 2a), with a corresponding decrease in the levels of XIAP (Fig. 2b). Moreover, we saw the appearance of the p30 XIAP fragment at 24 h post-treatment, which corresponded to the time point where the most significant increase in caspase-3 activity was observed.

### ARRY-520-induced apoptosis involves the activation of Caspase-2 but not the mitochondrial pathway

Our next objective was to determine the upstream signals involved in ARRY-520-induced apoptosis. Caspase-2 is a more recently described initiator caspase required in stress-induced apoptosis [18]. Thus, we determined caspase-2 activation in ARRY-520-treated Type II EOC cells using western blot analysis. Our results showed that ARRY-520 is able to induce caspase-2 activation in a time-dependent manner similar to that observed with the other caspases-9, -8, and -3 (Fig. 2b).

**Figure 3:**
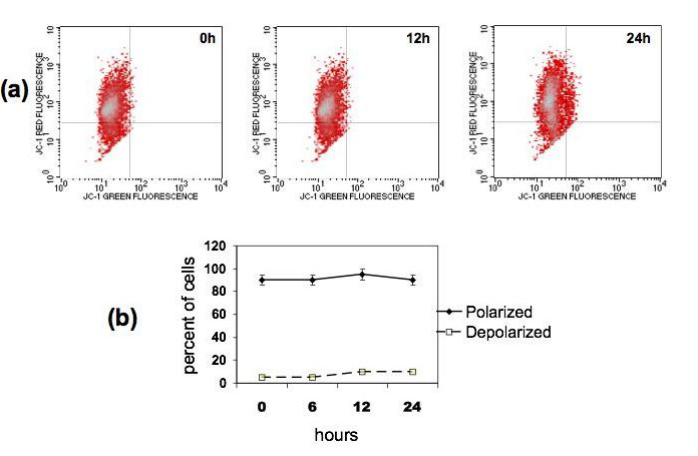
**ARRY-520 induces apoptosis independent of the mitochondrial pathway**. (a) Type II EOC cells were treated with 3 μM ARRY-520 for 12 and 24 hours, stained with JC-1 dye as described in the *Materials and Methods *section, and mitochondrial integrity was analyzed using Flow cytometry. (b) Graphical representation of the percentage of polarized and depolarized cells. Note that ARRY-520 does not induce mitochondrial depolarization. Results shown are obtained with CP70 cells. Similar results were observed with other cells tested.

Previous studies showed that caspase-2 could initiate apoptosis via three mechanisms. First, by direct action on mitochondrial membranes [19], second, by inducing mitochondrial depolarization through Bid [20], and third, by direct activation on effector caspases [21]. To further characterize ARRY-520-induced apoptosis, we next determined which of these pathways occur downstream of caspase-2. Western blot analysis of whole cell lysates showed that full-length Bid is maintained (Fig. 2b) and therefore is not activated. Furthermore, analysis of mitochondrial integrity showed that the mitochondria remain intact in ARRY-520-treated cells (Fig. 3a and 3b). These results suggest that ARRY-520-induced caspase-2 activation leads to the direct activation of effector caspases without the involvement of the mitochondria.

### ARRY-520 does not induce NF-κB activation and cytokine secretion in Type I EOC cells

ARRY-520 and Paclitaxel are both antimitotic agents but target different components of the mitosis machinery. Whereas Paclitaxel targets the microtubules directly, ARRY-520 targets the kinesin spindle protein.

**Figure 4:**
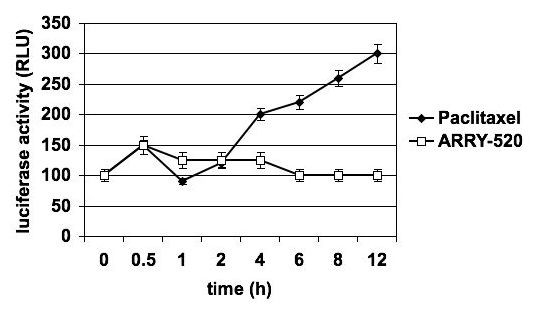
**Differential effect of ARRY-520 and Paclitaxel on NF-κB activation in Type I EOC cells**. Cells were transfected with a luciferase reporter plasmid activated by NF-κB and treated with either 3 μM ARRY-520 or 2 μM Paclitaxel. NF-κB activity was measured as luminescence. Data shown are for R182 cells. Similar results were obtained with other Type I EOC cells tested.

**Figure 5:**
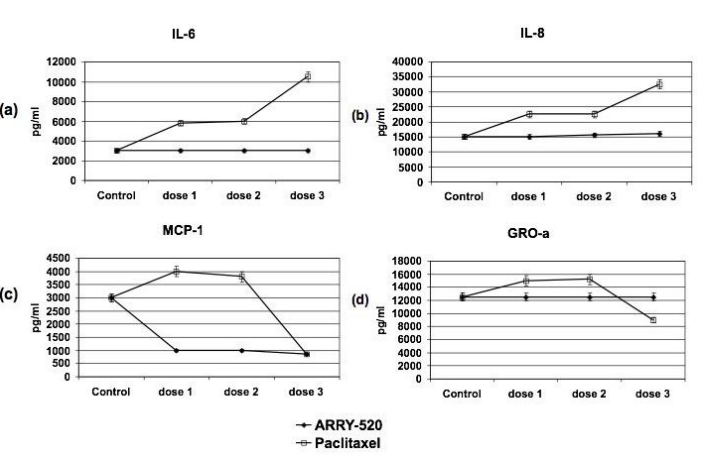
**Differential effect of ARRY-520 and Paclitaxel on cytokine profile in Type I EOC cells**. Cells were treated with ARRY-520 (0.03, 0.3, 3 μM) or Paclitaxel for (0.2, 2, 20 μM) for 48 hrs and levels of secreted cytokines/chemokines were determined using xMAP technology.

Recently, we reported that Paclitaxel, which is a known TLR-4 ligand, is able to activate NF-κB and induce the secretion of pro-inflammatory cytokines and chemokines in Type I EOC cells [4,5]. Thus, our next objective was to determine the effect of ARRY-520 on NF-κB and cytokine profile in this sub-group of EOC cells. As shown in Fig. 4, unlike Paclitaxel, ARRY-520 at the highest dose used (3 μM) does not induce NF-κB activation. In addition, ARRY-520 does not increase the secretion of pro-tumor cytokines IL-6, IL-8, and GRO-α (Fig. 5), which was previously seen with Paclitaxel treatment. Instead, ARRY-520 is able to down-regulate the constitutive MCP-1 secretion in these cells.

### ARRY-520 does not induce ERK1/2 phosphorylation in Type I EOC cells

**Figure 6:**
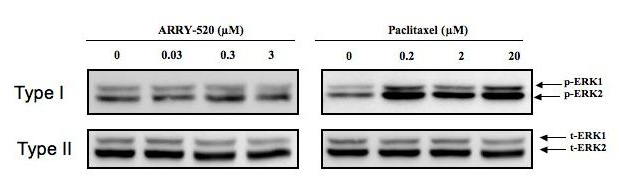
**Differential effect of ARRY-520 and Paclitaxel on ERK activation in Type I EOC cells**. Cells were treated with ARRY-520 (0.03, 0.3, 3 μM) or Paclitaxel for (0.2, 2, 20 μM) for 24 hrs and levels of phospho-ERK (p-ERK) and total ERK (t-ERK) weredetermined by Western blotting.

The extracellular signal-regulated kinase (ERK) pathway is involved in the regulation of cell proliferation, cell differentiation, and cell survival [22]. Physiological doses of Paclitaxel have been previously shown to induce a sustained phosphorylation of ERK 1/2 in human esophageal squamous cancer cells [23]. This is probably a compensatory survival response by the cancer cells to the drug treatment. Therefore, we evaluated the differential effect of Paclitaxel and ARRY-520 on the phosphorylation status of ERK 1/2 in Type I EOC cells. Paclitaxel, but not ARRY-520, induced the phosphorylation of ERK 1/2 (Fig. 6). Taken together, these results suggest that in Type I EOC cells and within the context of decreased cell viability, Paclitaxel is able to activate pro-survival pathways, which may lead to compensatory proliferation in the remaining viable cells. The activation of these pro-survival pathways was however, not observed with ARRY-520 treatment.

### ARRY-520 has comparable in vivo activity to Paclitaxel

**Figure 7:**
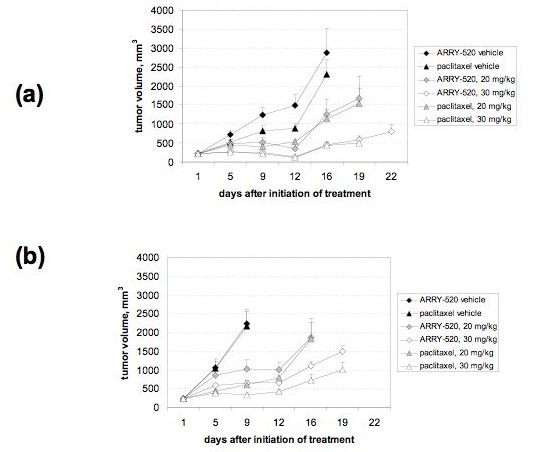
***In vivo *activity of ARRY-520 and Paclitaxel**. EOC tumors were established s.c. in female nude mice and treatments were given as described in the *Materials and Methods *section. Tumor size was determined by caliper measurements. (a) A2780 xenograft model and (b) tumors established from a primary culture of EOC cells.

Our final objective was to determine the activity of ARRY-520 in an EOC mice xenograft model. Thus, we established a subcutaneous (s.c.) model in nude mice using A2780, an established EOC cell line, and R182, a primary culture isolated from patient's ascites (Type II and Type I, respectively). The anti-tumor activitiy of ARRY-520 and Paclitaxel was then determined as described in the Methods section. In this animal model, the results confirmed our *in vitro *observation that the compounds demonstrate equivalent activity against ovarian cancer cells. Both compounds induced a decrease in tumor kinetics in a dose-dependent manner (Fig. 7a and 7b).

## Discussion

We demonstrate in this study that the KSP inhibitor, ARRY-520, has similar anti-tumor activity in EOC cells compared to Paclitaxel. More importantly though, unlike Paclitaxel, ARRY-520 does not activate NF-κB and does not induce secretion of pro-tumor cytokines in Type I EOC cells. Therefore, ARRY-520 may represent an alternative to Paclitaxel in this subgroup of EOC cells.

KSP is a microtubule-associated motor protein, which is essential for centrosome separation, formation of a bipolar mitotic spindle, and proper segregation of sister chromatids during mitosis [24]. Inhibition of KSP forms monopolar mitotic spindles and arrests cells at mitosis, which leads to cell death [25,26]. KSP inhibitors have been shown to exhibit antitumor activity and are currently in clinical trials [7,9]. Because KSP localizes to mitotic microtubules, KSP inhibitors function exclusively during mitosis and are therefore selective to mitotic cells. Indeed, KSP inhibitors are shown to spare post mitotic neurons and thus do not cause peripheral neuropathy, which is a major side effect observed in Paclitaxel treatment [9]. In the present study, we showed an additional advantage for the use of the KSP inhibitor ARRY-520 over Paclitaxel, specifically in Type I EOC cells.

In the subgroup of EOC cells with a functional TLR-4/MyD88/NF-κB pathway, Paclitaxel treatment leads to proliferation and NF-κB activation [4,14]. The activation of NF-κB is a major component in cancer initiation and progression [27] and plays a central role in the control of apoptosis, cell proliferation, and survival [28,29]. Animal models have further supported the link between NF-κB activation and cancer progression [30]. The demonstration that Paclitaxel can bind to TLR4 [31] and therefore activate NFκB could explain why we observe tumor growth during Paclitaxel treatment [4]. The absence of NFκB activation after ARRY-520 treatment suggests that ARRY-520 may be a better treatment option in patient with Type I EOC cells.

Another important aspect associated with NF-κB activation is the potential effect on the immune system. We showed previously that in Type I EOC cells, Paclitaxel treatment is able to induce the secretion of the pro-inflammatory cytokines IL-6, IL-8, MCP-1, and GROα [5,14]. All of these cytokines have been shown to directly affect cancer cell survival and growth [32,33] and also have implications in the resulting immune response. Indeed, our group has shown that the secretion of these cytokines by the Type I EOC cells is able to modulate the type of cytokines produced by the monocyte-like THP-1 cell line [34]

It was noted that the mice with xenografts obtained from either the Type I or Type II cell lines responded equally to both compounds. These results did not reflect those seen *in vitro *where Type I EOC cells are more resistant to treatment. Our group recently reported the identification and characterization of the ovarian cancer stem cells using the cell surface marker, CD44 [14]. In this report, we showed that CD44+ cells represent the specific cell population that has a functional TLR-4/MyD88/NF-κB pathway. Indeed injection of R182 cells in mice (which is > 90% CD44+ by flow cytometry pre-injection) resulted in s.c. tumors containing < 10% CD44+ positive cells [14]. The differentiation of the R182 cells from Type I to Type II *in vivo *may explain the equivalent chemoresponse observed from the two xenograft models.

It is important to emphasize that this response induced by Paclitaxel is not observed in all EOC cells, but is limited to a specific sub-group, the Type I EOC cells.

In summary, ARRY-520 may represent an alternative to Paclitaxel in Type I EOC cells. This suggests the importance of identifying the molecular phenotype of the tumor prior to the initiation of therapy.

## Conclusion

Administration of Paclitaxel to patients with high percentage Type I cancer cells could have detrimental effects due to Paclitaxel-induced enhancement of NF-κB and ERK activities and cytokine production (e.g. IL-6), which promote chemoresistance and tumor progression. ARRY-520 has similar anti-tumor activity in EOC cells as that of Paclitaxel. However, unlike Paclitaxel, it does not induce these pro-tumor effects in Type I cells. Therefore, the KSP inhibitor ARRY-520 may represent an alternative to Paclitaxel in this subgroup of EOC patients.

## Abbreviations

EOC: epithelial ovarian cancer cell; KSP: kinesin spindle protein; NF-κB: nuclear factor κB; XIAP: X-linked inhibitor of apoptosis protein; JC-1: 5,5',6,6'-tetrachloro-1,1',3,3'-tetraethyl-benzamidazolocarbocyanin iodide

## Competing interests

KK, YX, ET, GM, and AA do not have competing interests. RW is an employee of Array Biopharma.

## Authors' contributions

KK and YX performed cell viability assays, western blots, and luciferase assays. ET performed the mitochondrial depolarization assay. RW performed the in vivo experiments. GM participated in the design of the study and helped to draft the manuscript. AA participated in the design, analysis, and coordination of the study and the final drafting of the manuscript. All authors have read and approved the final manuscript.
